# Supplementary material for: The phytopathogenic fungus Sclerotinia sclerotiorum detoxifies plant glucosinolate hydrolysis products via an isothiocyanate hydrolase
Source: Nat Commun. 2020 Jun 18;11:3090. doi: 10.1038/s41467-020-16921-2 (PMC7303113; doi:10.1038/s41467-020-16921-2)
Supplement: Supplementary file 1 — Supplementary Information [file 41467_2020_16921_MOESM1_ESM.pdf]

Supplementary Information

The phytopathogenic fungus *Sclerotinia sclerotiorum* detoxifies plant glucosinolate hydrolysis products via an isothiocyanate hydrolase

Chen, J. et al.

Supplementary Figures 1-12

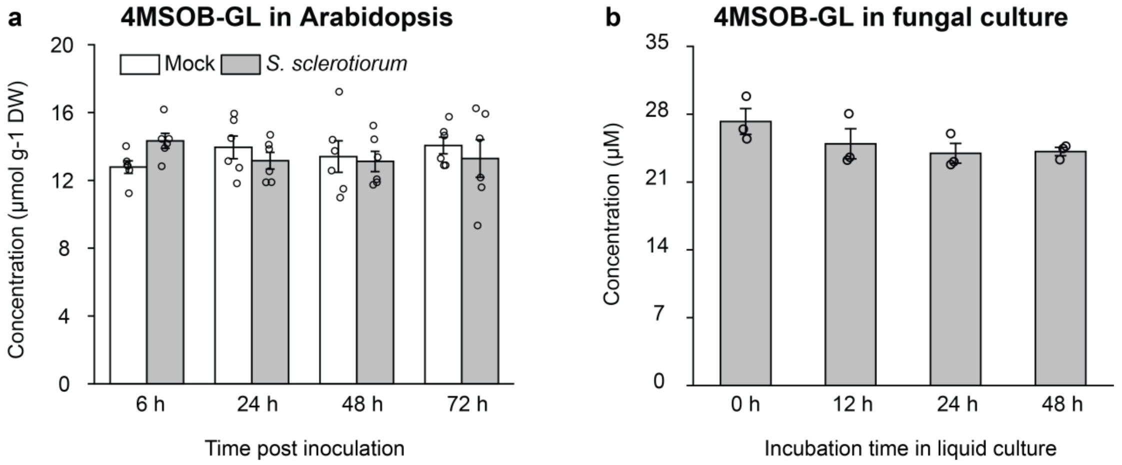

Supplementary Figure 1. 4MSOB-GL is not reduced by *S. sclerotiorum* in Arabidopsis leaves and in liquid culture. (a) 4MSOB-GL in Arabidopsis leaves inoculated with *S. sclerotiorum*. An agar plug without fungus was used in mock inoculations. Data represent mean ± SEM (n = 6 independent inoculated plants) and were analyzed by Kruskal-Wallis rank sum test (p = 0.56). **(b)** Pure 4MSOB-GL (25 µM) was supplied to fungal cultures and its concentration was monitored at multiple time points up to 48 h. Data represent mean ± SEM (n = 3 independent fungal cultures) and were analyzed by one-way ANOVA (p = 0.25). 4MSOB-GL, 4-methylsulfinylbutyl glucosinolate; DW, dry weight. Source data are provided as a Source Data file.

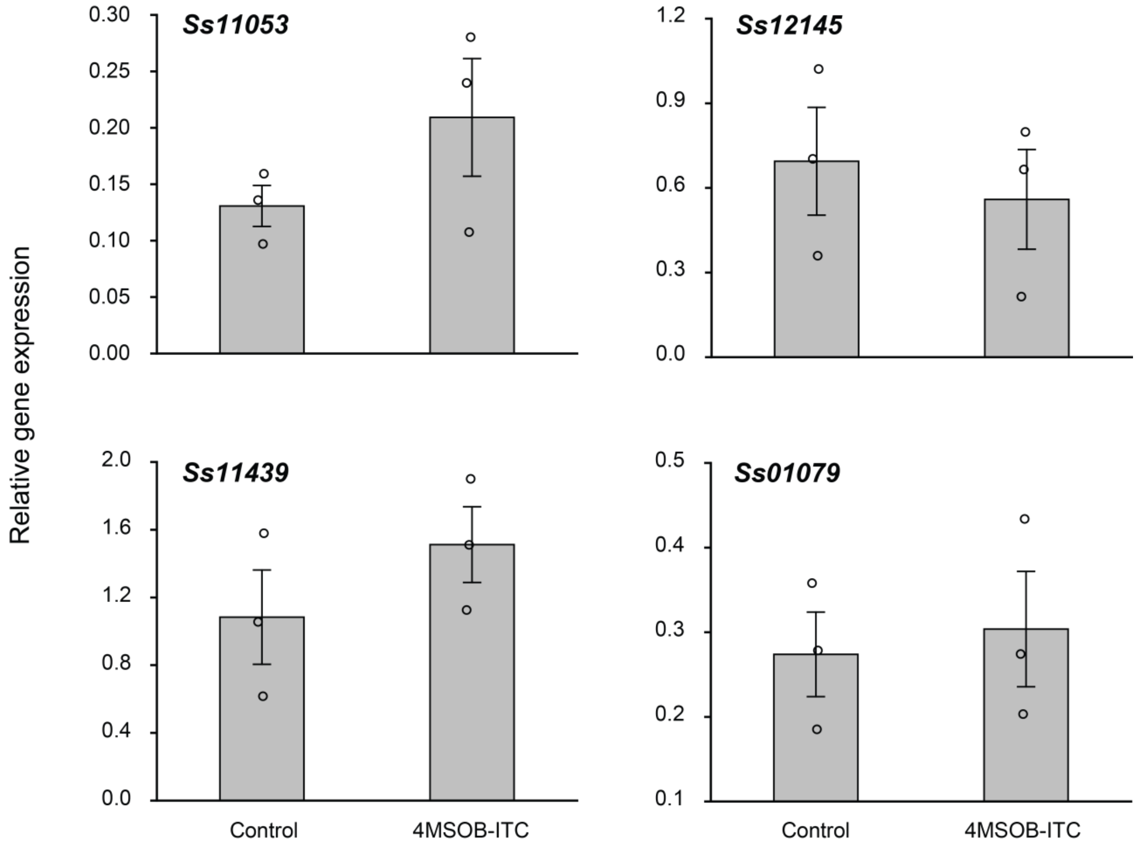

Supplementary Figure 2. Expression of candidate genes other than *Ss12040* is not induced by 4MSOB-ITC. The fungal culture medium was supplemented with 4MSOB-ITC (25 µM), and only ethanol was added to the control medium. The mycelium of *S. sclerotiorum* was harvested 30 min post inoculation and gene expression was determined by qRT-PCR. Data represent mean ± SEM (n = 3 independent fungal cultures) and were analyzed by a two-tailed Student's *t*-test (p = 0.66 for *Ss11053*; p = 0.23for *Ss12145*; p = 0.63for *Ss11439* and p = 0.30 for *Ss01079*). 4MSOB-ITC, 4-methylsulfinylbutyl isothiocyanate. Source data are provided as a Source Data file.

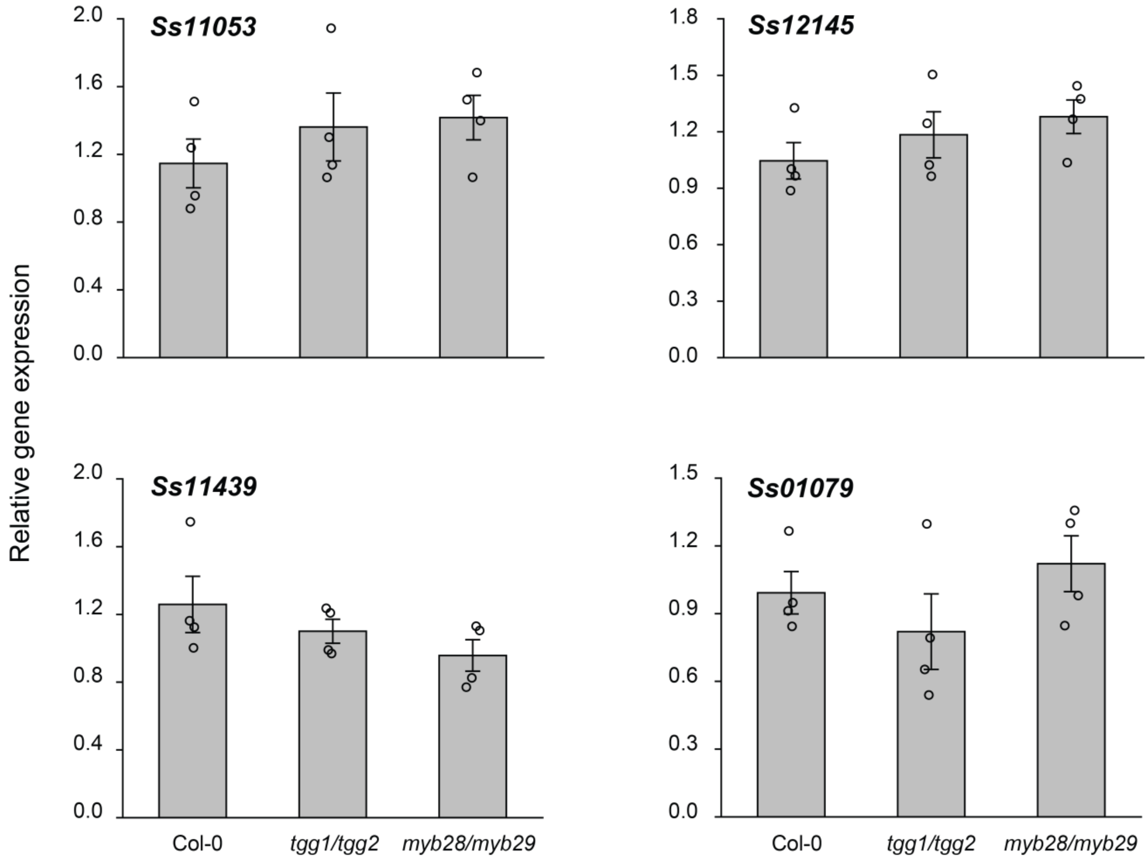

Supplementary Figure 3. Other *SaxA* candidate genes have unaltered expression on ITC-deficient *A. thaliana*. Data represent mean ± SEM (n = 4 independent inoculated plants) and were analyzed by one-way ANOVA (p = 0.48 for *Ss11053*; p = 0.32 for *Ss 12145*; p = 0.25 for *Ss11439* and p = 0.32 for *Ss01079*). Source data are provided as a Source Data file.

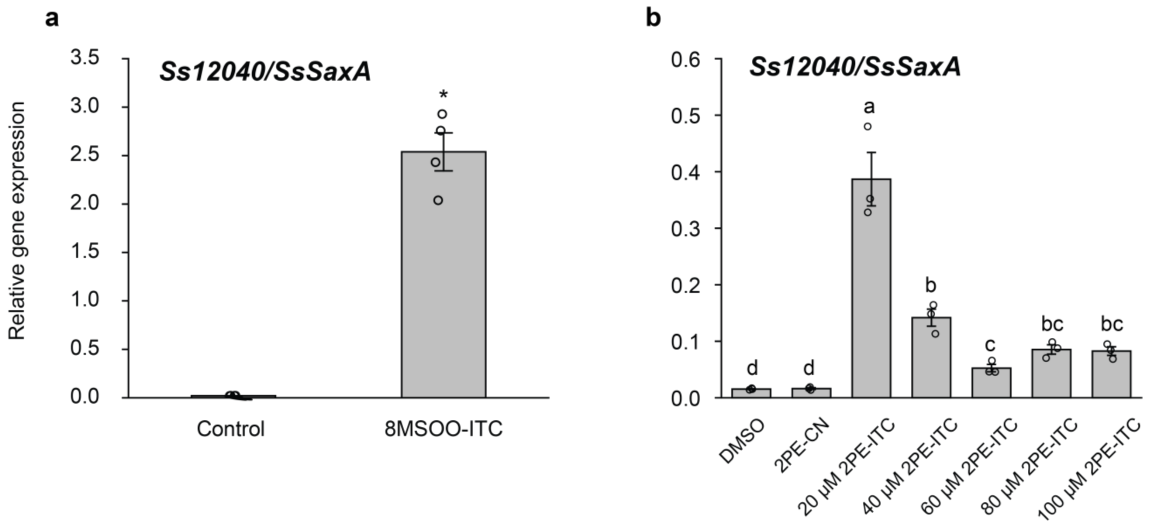

Supplementary Figure 4. Expression of *SsSaxA* is induced by 8MSOO-ITC and 2PE-ITC. (a) Transcript abundances of *SsSaxA* gene in *S. sclerotiorum* after incubation with 8MSOO-ITC (25 µM) for 30 min or with only ethanol being added to the control medium. Data represent mean ± SEM (n = 4 independent fungal cultures) and were analyzed by a two-tailed Mann-Whitney rank sum test (* $p < 0.05$). **(b)** Expression of *SsSaxA* after fungal mycelium was exposed to 2PE-ITC at different concentrations, with DMSO and 2PE-CN being added as controls. Data represent mean ± SEM (n = 3 independent fungal cultures) and were analyzed by one-way ANOVA ($p < 0.001$) followed by Tukey's post-hoc test. Different letters above the bars indicate significant differences at $p < 0.05$. 8MSOO-ITC, 8-methylsulfinyloctyl isothiocyanate; 2PE-CN, 3-phenylpropanenitrile; DMSO, dimethyl sulfoxide; 2PE-ITC, 2-phenylethyl isothiocyanate. Source data are provided as a Source Data file.

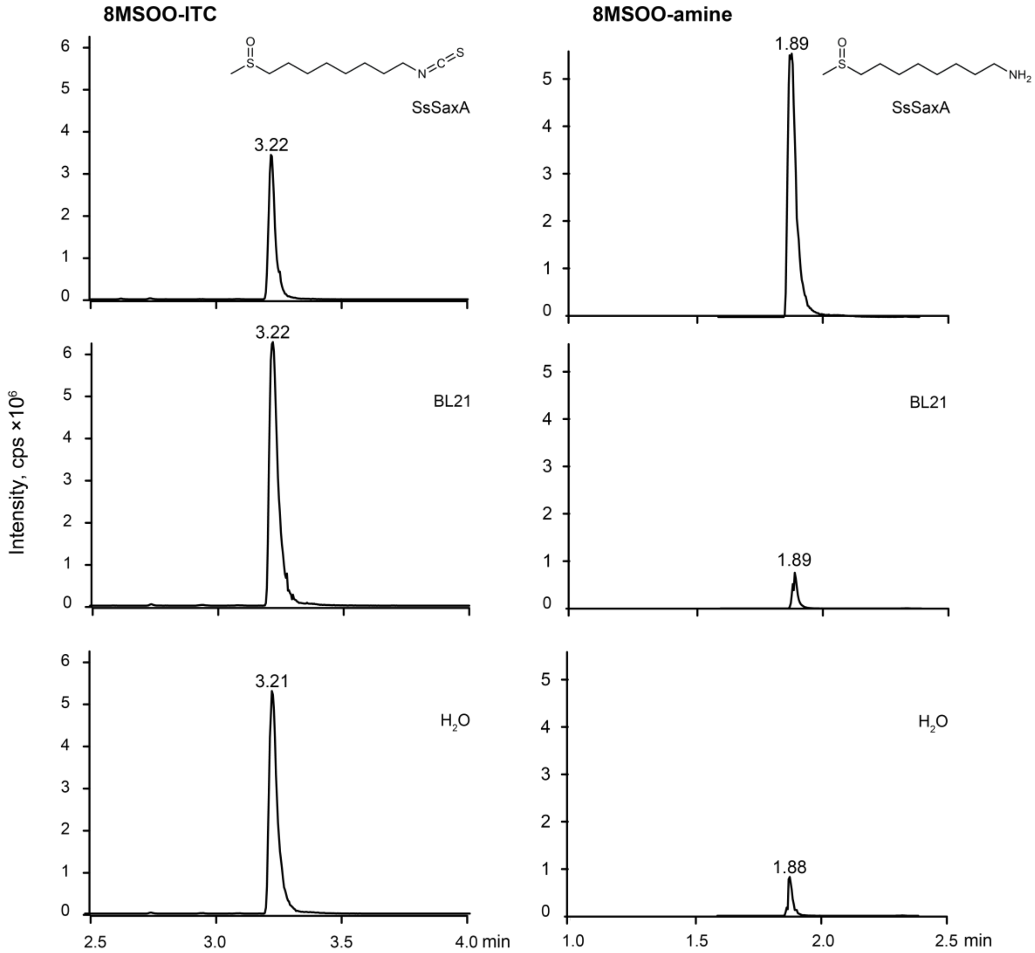

Supplementary Figure 5. Heterologously expressed SsSaxA exhibits activity towards 8MSOO-ITC. Crude protein from *E. coli* BL21 and water were used as controls. 8MSOO-ITC and -amine, 8-methylsulfinyloctyl isothiocyanate and amine.

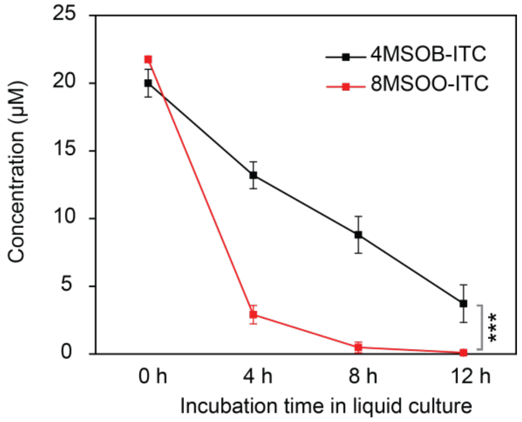

Supplementary Figure 6. *S. sclerotiorum* degrades 8MSOO-ITC in the medium more rapidly than 4MSOB-ITC. 25 µM 4MSOB-ITC or 8MSOO-ITC was added initially to each fungal culture. Data represent mean ± SEM (n = 3 independent fungal cultures) and were analyzed by one-way ANCOVA (*** $p < 0.001$). 4MSOB- and 8MSOO-ITC, 4-methylsulfinylbutyl and 8-methylsulfinyloctyl isothiocyanate. Source data are provided as a Source Data file.

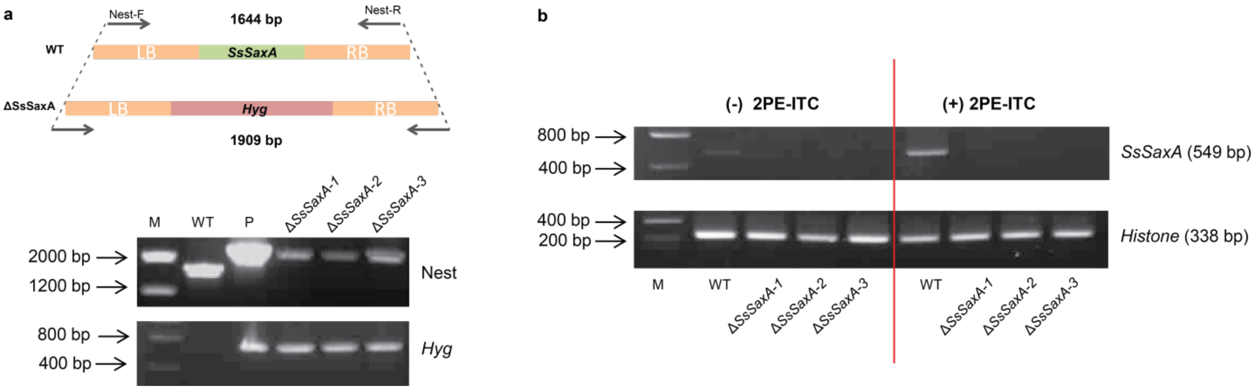

Supplementary Figure 7. Confirmation of *SsSaxA* gene replacement in Δ*SsSaxA* mutants. (a) PCR verification of genomic DNA from WT and Δ*SsSaxA* mutants with nested primers and hygromycin gene primers. **(b)** Semi-quantitative reverse transcriptase-PCR of *SsSaxA* gene in WT and Δ*SsSaxA* mutants induced with 2PE-ITC. DMSO was supplied as control. M, low mass DNA marker; WT, wild-type fungus; P, pXEH replacement vector (positive control); 2PE-ITC, 2-phenylethyl isothiocyanate. Δ*SsSaxA* mutations were independently confirmed twice. Source data are provided as a Source Data file.

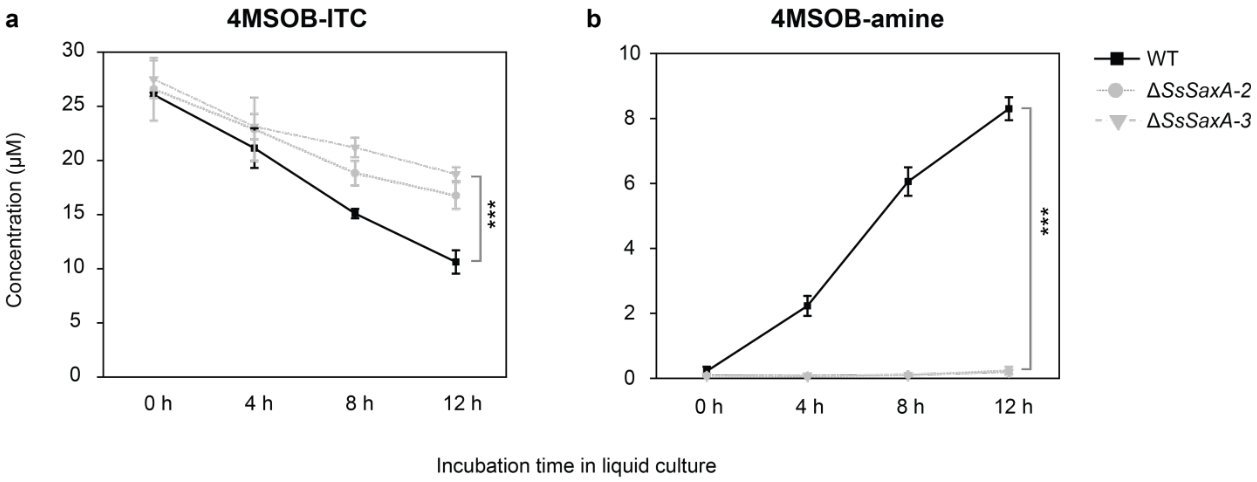

Supplementary Figure 8. Δ*SsSaxA-2* and -*3* mutants degrade 4MSOB-ITC less efficiently. Quantification of **(a)** 4MSOB-ITC and **(b)** 4MSOB-amine in WT and Δ*SsSaxA* fungal cultures over a time course. 25 µM 4MSOB-ITC was used for each fungal culture initially. Data represent mean ± SEM (n = 3 independent fungal cultures) and were analyzed by one-way ANCOVA (*** $p < 0.001$ for WT/ Δ*SsSaxA*-2 and WT/ Δ*SsSaxA-3*). WT, wild-type fungus; 4MSOB-ITC and -amine, 4-methylsulfinylbutyl isothiocyanate and amine. Source data are provided as a Source Data file.

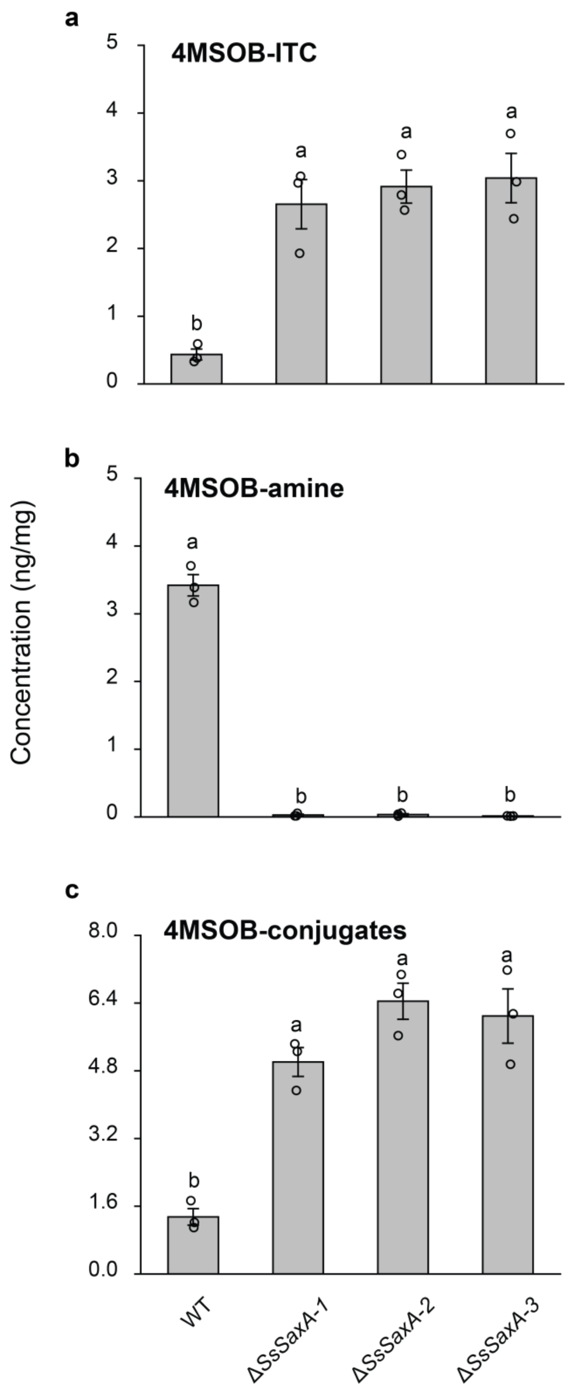

Supplementary Figure 9. 4MSOB-ITC is metabolized differently in WT and Δ*SsSaxA* mycelia. Quantification of **(a)** 4MSOB-ITC, its degradation products **(b)** 4MSOB-amine and **(c)** 4MSOB-conjugates per mg fresh mycelium of WT and Δ*SsSaxA* mutants. Data represent mean ± SEM (n = 3 independent fungal cultures) and were analyzed by one-way ANOVA ($p < 0.001$) followed by a Tukey's post-hoc test. Different letters above the bars indicate significant differences at $p < 0.05$. 4MSOB-ITC and -amine, 4-methylsulfinylbutyl isothiocyanate and amine; 4MSOB-conjugates include 4MSOB-ITC glutathione

conjugate, cysteinylglycine conjugate, cysteine conjugate and *N*-acetylcysteine conjugate. FW, fresh weight. Source data are provided as a Source Data file.

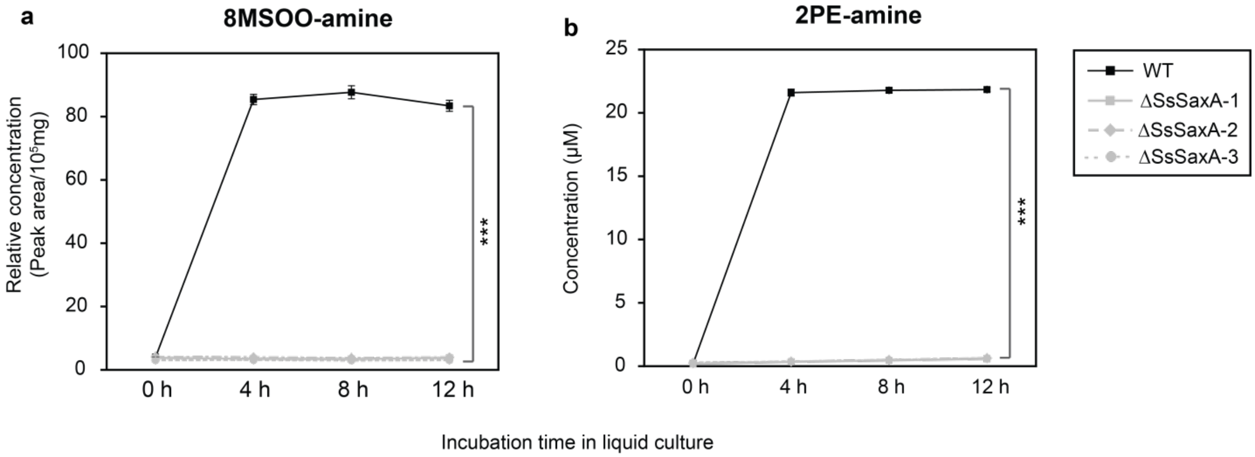

Supplementary Figure 10. 8MSOO- and 2PE-amines accumulate only in WT fungal cultures. 25 μM 8MSOO-ITC **(a)** or 2PE-ITC **(b)** was used for each fungal culture initially. Data represent mean ± SEM (n = 3 independent fungal cultures) and were analyzed by one-way ANCOVA (*** $p < 0.001$ for WT/ Δ*SsSaxA-1*, WT/ Δ*SsSaxA-2* and WT/ Δ*SsSaxA-3*). WT, wild-type fungus; 8MSOO- and 2PE-amine, 8-methylsulfinyloctyl and 2-phenylethyl amine. Source data are provided as a Source Data file.

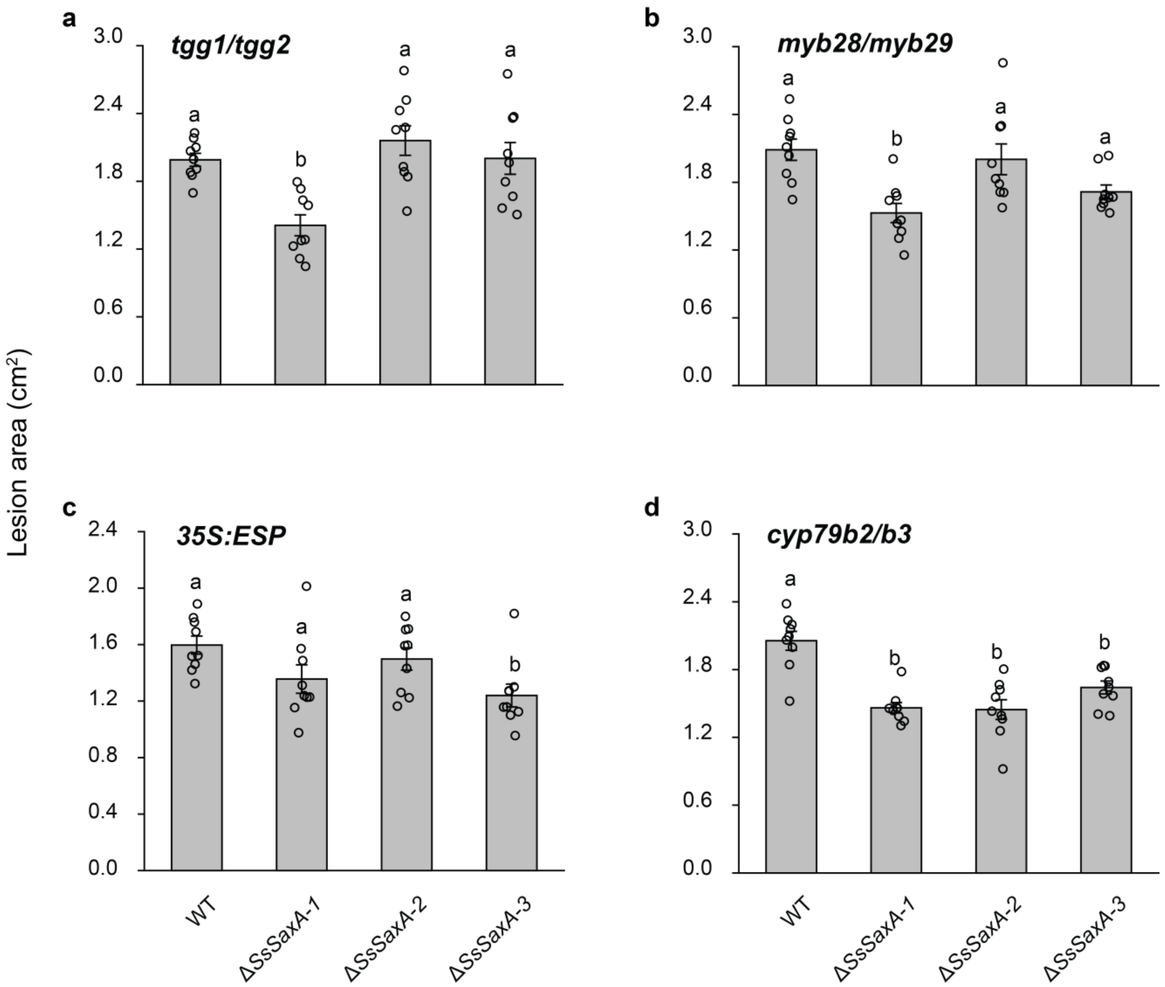

Supplementary Figure 11. Pathogenicity of Δ*SsSaxA* mutants is partially recovered on ITC deficient *A. thaliana* mutants. Comparison of lesion areas caused by WT strain and Δ*SsSaxA* mutants 24 hpi on leaves of the *A. thaliana* **(a)** aliphatic ITC deficient mutants *tgg1/tgg2*, **(b)** the aliphatic GL deficient mutant *myb28/myb2*9, **(c)** an ESP overexpressing line *35S:ESP* and **(d)** an indolic GLs deficient mutant *cyp79b2/b3*. All data represent mean ± SEM (n = 9 inoculated leaves from separate plants) and were analyzed by one-way ANOVA ($p < 0.05$) followed by a Tukey's post-hoc test. Different letters above the bars indicate significant differences at $p < 0.05$. Source data are provided as a Source Data file.

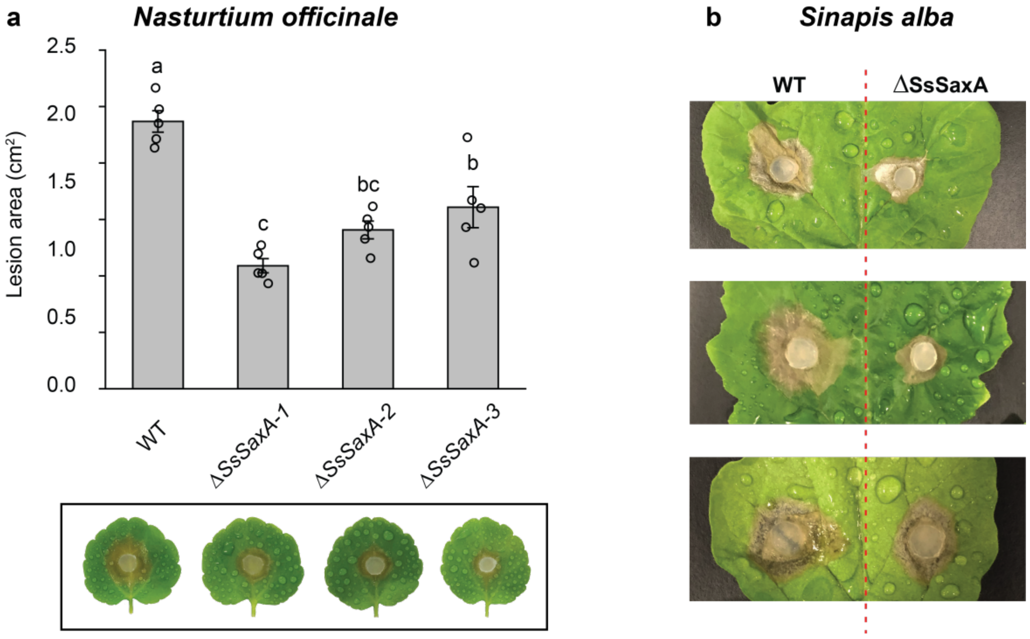

Supplementary Figure 12. Pathogenicity of Δ*SsSaxA* mutants on *Brassica* plants is significant reduced. (a) Quantification of lesion area caused by the WT fungus and Δ*SsSaxA* mutants on *Nasturtium officinale* 24 hpi. Data represent mean ± SEM (n = 5 inoculated leaves from separate plants) and were analyzed by one-way ANOVA ($p < 0.001$) followed by a Tukey's post-hoc test. Different letters above the bars indicate significant differences at $p < 0.05$. **(b)** Image of *Sinapis alba* leaves inoculated with WT and Δ*SsSaxA* fungi at 24 hpi. Inoculation of *N. officinale* and *S. alba* was repeated once. Source data are provided as a Source Data file.
