# Supplementary material for: The phytopathogenic fungus Sclerotinia sclerotiorum detoxifies plant glucosinolate hydrolysis products via an isothiocyanate hydrolase
Source: Nat Commun. 2020 Jun 18;11:3090. doi: 10.1038/s41467-020-16921-2 (PMC7303113; doi:10.1038/s41467-020-16921-2)
Supplement: Supplementary file 3 — Reporting Summary [file 41467_2020_16921_MOESM3_ESM.pdf]

nature research

Corresponding author(s): Daniel Giddings Vassão & Almuth Hammerbacher

Last updated by author(s): May 10, 2020

Reporting Summary

Nature Research wishes to improve the reproducibility of the work that we publish. This form provides structure for consistency and transparency in reporting. For further information on Nature Research policies, see our Editorial Policies and the Editorial Policy Checklist.

Statistics

For all statistical analyses, confirm that the following items are present in the figure legend, table legend, main text, or Methods section.

n/a	Confirmed	
☐	☒	The exact sample size (*n*) for each experimental group/condition, given as a discrete number and unit of measurement
☐	☒	A statement on whether measurements were taken from distinct samples or whether the same sample was measured repeatedly
☐	☒	The statistical test(s) used AND whether they are one- or two-sided<br>*Only common tests should be described solely by name; describe more complex techniques in the Methods section.*
☒	☐	A description of all covariates tested
☐	☒	A description of any assumptions or corrections, such as tests of normality and adjustment for multiple comparisons
☐	☒	A full description of the statistical parameters including central tendency (e.g. means) or other basic estimates (e.g. regression coefficient) AND variation (e.g. standard deviation) or associated estimates of uncertainty (e.g. confidence intervals)
☐	☒	For null hypothesis testing, the test statistic (e.g. *F*, *t*, *r*) with confidence intervals, effect sizes, degrees of freedom and *P* value noted<br>*Give P values as exact values whenever suitable.*
☒	☐	For Bayesian analysis, information on the choice of priors and Markov chain Monte Carlo settings
☒	☐	For hierarchical and complex designs, identification of the appropriate level for tests and full reporting of outcomes
☒	☐	Estimates of effect sizes (e.g. Cohen's *d*, Pearson's *r*), indicating how they were calculated

Our web collection on statistics for biologists contains articles on many of the points above.

Software and code

Policy information about availability of computer code

Data collection	Photoshop CS5; Anayst 2.1 (AB-Sciex), Bio-Rad CFX Real-Time PCR Systems
Data analysis	Mega5.05; PhyML v. 3.0; SigmaPlot12.0; SPSS 17.0; R3.1.1; Adobe Illustrator CS5; FigTree v1.4.2

For manuscripts utilizing custom algorithms or software that are central to the research but not yet described in published literature, software must be made available to editors and reviewers. We strongly encourage code deposition in a community repository (e.g. GitHub). See the Nature Research guidelines for submitting code & software for further information.

Data

Policy information about availability of data

All manuscripts must include a data availability statement. This statement should provide the following information, where applicable:

- Accession codes, unique identifiers, or web links for publicly available datasets
- A list of figures that have associated raw data
- A description of any restrictions on data availability

The data supporting the findings of this study are available within the paper, its supplementary information files, and the source data file. Accession numbers for all proteins referred to this manuscript are given in Data availability section and are accessible from NCBI (https://www.ncbi.nlm.nih.gov/).

Field-specific reporting

Life sciences study design

All studies must disclose on these points even when the disclosure is negative.

Sample size	For fungal growth assays with different compounds we had 3-4 biological replicates, the variations were small between replicates and differences were significant between groups. Experiments were repeated one more time to confirm the results. For the in vivo plant pathogenicity assay, each plant had 4-5 inoculated leaves as one replicate, we had 6 plants for each treatment as replicates. In vitro pathogenicity assays were performed with 6-9 independent leaves and the whole experiment was repeated one more time.
Data exclusions	No data were excluded.
Replication	In vitro pathogenicity and fungal growth assays were performed twice and had consistent results. Tests of in vitro induction of SsSaxA expression were performed twice.
Randomization	Fungal cultures were randomly selected for different treatments; Arabidopsis plants from the same individual batches were randomly used in each experiment for mock, WT S. sclerotiorum and mutant fungi inoculation.
Blinding	The chemical and molecular measurements were automatically processed by software Analyst 2.1 and BioRad CFX Real-Time PCR Systems, and resulting extracted data were all directly further analysed without data points being selected or excluded. Fungal radial growth with different compounds was measured by a person not involved in this project for confirmation.

Reporting for specific materials, systems and methods

We require information from authors about some types of materials, experimental systems and methods used in many studies. Here, indicate whether each material, system or method listed is relevant to your study. If you are not sure if a list item applies to your research, read the appropriate section before selecting a response.

Materials & experimental systems

n/a	Involved in the study
☒	☐ Antibodies
☒	☐ Eukaryotic cell lines
☒	☐ Palaeontology and archaeology
☒	☐ Animals and other organisms
☒	☐ Human research participants
☒	☐ Clinical data
☒	☐ Dual use research of concern

Methods

n/a	Involved in the study
☒	☐ ChIP-seq
☒	☐ Flow cytometry
☒	☐ MRI-based neuroimaging
